# Maximal Genetic Code Symmetry Is a Physicochemical Purine–Pyrimidine Symmetry Language for Transcription and Translation in the Flow of Genetic Information from DNA to Proteins

**DOI:** 10.3390/ijms25179543

**Published:** 2024-09-02

**Authors:** Marija Rosandić, Vladimir Paar

**Affiliations:** 1 Department of Internal Medicine, University Hospital Centre Zagreb, (Ret.), 10000 Zagreb, Croatia; 2 Croatian Academy of Sciences and Arts, 10000 Zagreb, Croatia; paar@hazu.hr; 3 Physics Department, Faculty of Science, University of Zagreb, 10000 Zagreb, Croatia

**Keywords:** genetic code symmetry, Supersymmetry Genetic Code table, Standard Genetic Code table, transcription, translation, wobble hypothesis, DNA symmetry

## Abstract

Until now, research has not taken into consideration the physicochemical purine–pyrimidine symmetries of the genetic code in the transcription and translation processes of proteinogenesis. Our Supersymmetry Genetic Code table, developed in 2022, is common and unique for all RNA and DNA living species. Its basic structure is a purine–pyrimidine symmetry net with double mirror symmetry. Accordingly, the symmetry of the genetic code directly shows its organisation based on the principle of nucleotide Watson–Crick and codon–anticodon pairing. The maximal purine–pyrimidine symmetries of codons show that each codon has a strictly defined and unchangeable position within the genetic code. We discovered that the physicochemical symmetries of the genetic code play a fundamental role in recognising and differentiating codons from mRNA and the anticodon tRNA and aminoacyl-tRNA synthetases in the transcription and translation processes. These symmetries also support the wobble hypothesis with non-Watson–Crick pairing interactions between the translation process from mRNA to tRNA. The Supersymmetry Genetic Code table shows a specific arrangement of the second base of codons, according to which it is possible that an anticodon from tRNA recognises whether a codon from mRNA belongs to an amino acid with two or four codons, which is very important in the purposeful use of the wobble pairing process. Therefore, we show that canonical and wobble pairings essentially do not lead to misreading and errors during translation, and we point out the role of physicochemical purine–pyrimidine symmetries in decreasing disorder according to error minimisation and preserving the integrity of biological processes during proteinogenesis.

## 1. Introduction

In the first half of the 20th century, Einstein and Schrödinger placed the symmetry principle as the primary feature of nature [1,2,3]. The maximal realisation of Einstein’s symmetry principle can be extended to biological processes. Namely, symmetries decrease disorder and lead to error minimisation. After Nirenberg’s discovery in 1961 that codons code the natural amino acids [4], a challenge has been to find the optimal symmetry of the genetic code table. In 1968, Crick proposed a solution in the form of the Universal Genetic Code table [5], which holds, to this day, a central position in all areas of biology and genetics. The Universal Genetic Code table consists of 61 codons, which are the fundamental structure for proteinogenesis and are assigned to a specific amino acid, and three terminating stop signals. The Universal Genetic Code table is given in 4 × 4 boxes with four codons in each box. The codons are positioned alphabetically in a horizontal and vertical array of U-C-A-G bases. The role of the third base of codons was ignored in the search for genetic code symmetries because the U-C-A-G array was the same in all boxes. As such, the codons in each box are differentiated only according to the first two bases.

However, symmetries based on the alphabetical principle do not exist in nature, and the Universal Genetic Code table and all other known genetic code tables built on the double direction of the U-C-A-G principle suffer from an inability to exhibit the complete purine–pyrimidine symmetry of codons. Symmetries were investigated in more than one hundred variations of codes, in circular, triangular, rectangular, torus and binary transformation of nucleotides in codons, and with respect to the polar requirement, but the results showed only a partial solution related to the physicochemical properties of the code. In the meantime, more than 30 slightly alternative variants of the nuclear and mitochondrial genetic codes were discovered, and the Universal Genetic Code table was given a new name—the Standard Genetic Code (SGC). Discovering alternative variants of the symmetrical genetic codes presents a challenge for scientists to this day, which continues to be pursued in order for us to understand the origin and evolution of the genetic code.

In 2022, we developed the Supersymmetry Genetic Code (SSyGC) table (see Section 4), with multifaceted physicochemical symmetries of the genetic code and the core purine–pyrimidine symmetry net at the central position, common for all RNA and DNA living species [6]. Our analysis of the genetic code table, based on the purine–pyrimidine symmetry properties, allowed for a fundamental comprehension of the genetic code structure. Within the physicochemical laws, the purine–pyrimidine symmetry net ensures a strictly defined position of each codon in the genetic code and, in this way, simultaneously ensures integrity and unchangeability during evolution.

Our “symmetry-based theory of the genetic code” with the SSyGC table also supports the role of quadruplet symmetries in the DNA molecules of each living species, as well the role of our trinucleotide/codon classification. One reviewer of our article with this development [7] commented on the SSyGC table as very interesting but related only to the genetic code, representing a kind of puzzle solution without broader significance. Their comment encouraged us to prove whether these symmetries have a role in the processes of transcription and translation during proteinogenesis. That was a challenge for us, and the results are presented in this paper. The goal of this investigation was to discover the role of genetic code symmetries in the flow of genetic information from DNA to proteins in the transcription and translation processes.

## 2. Results

### 2.1. The Role of Identical Symmetries of DNA and mRNA in Transcription

Transcription is a process in which a DNA molecule is copied to an mRNA and carries information that is needed for protein synthesis. In eukaryotes, it occurs in the cell nucleus. The strand of DNA that contains the genes is the sense strand, and the mRNA as a single strand produces a copy of the sense strand via transcription. RNA splicing is a process that enables the creation of the primary DNA transcript. It contains the intervening sequences with exons and introns from the eukaryotic gene under the influence of a spliceosome as a protein complex, which cuts out introns. Aligning only exons, the mature mRNA is created, which contains uracil instead of thymine, while the other three bases (adenine, cytosine, guanine) remain identical. 

**Figure 1:**
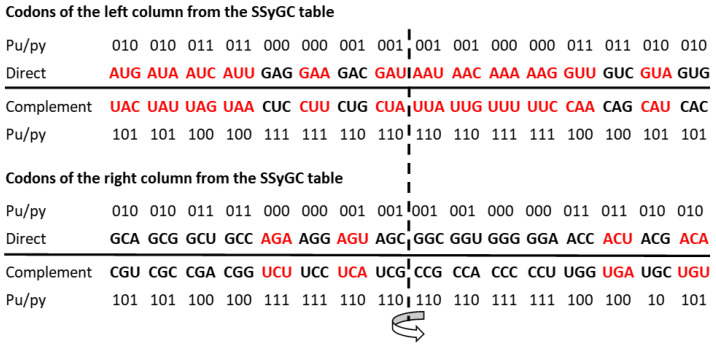
A direct transformation of the SSyGC table into the DNA molecule with the codons from direct boxes for one DNA strand and the codons from complement boxes for the opposite DNA strand. A+T-rich codons (red) from the first column of code and C+G-rich codons (black) from the second column are in a purine–purine and pyrimidine–pyrimidine relationship between bases (A↔G, U↔C). There is also a purine–pyrimidine double mirror symmetry net between both the horizontal and vertical halves of the DNA molecule. In each column, there is a position of codons with an identical configuration, e.g., AAA, UUU, GGG, CCC or GUG, CAC, ACA, UGU.

In this way, the same fundamental symmetry based on the principle of Watson–Crick A↔U and C↔G base pairing (synonyms: direct–complement, sense–antisense and 5′3′ codon–3′5′ anticodon) is transferred from the DNA molecule and the genetic code to mRNA and to the cognate tRNA. This is in accordance with the direct transformation of the same ordering of the complementary pairs of codons from the SSyGC table into the DNA molecule (Figure 1). The maximal symmetrical position of similar codons, for example, AAA–UUU and GGG–CCC or AUA–UAU and GCG–CGC, is in the same column because of the double mirror purine–pyrimidine symmetry net of the SSyGC table (Figure 1 and see Section 4).

Single-strand viruses, as the simplest living species, have no symmetries based on the principle of Watson–Crick pairing of nucleotides because the second strand is missing. However, single-strand viruses have an identical SSyGC table to all other DNA living species. Our genetic code is structured with symmetrical exchange in the pairs of direct–complement codon boxes (see Section 4). As such, the genetic code is not only a code for natural amino acids but also the code for the direction of evolution from RNA to DNA species based on the principle of Watson–Crick pairing.

### 2.2. Translation Is Based on Genetic Code Symmetries

The essential processes of translation are deciphering mRNA codons and the attachment of the appropriate amino acid to the specified tRNA in order to form a growing polypeptide chain. tRNA is a bridging molecule that leads to the identification of enzymes responsible for linking the nucleotides of mRNA and aminoacyl-tRNA synthetases (aaRSs). aaRSs play a crucial role in translation as an essential and universally distributed family of enzymes, pairing tRNA with their cognate amino acids in order to decode mRNA according to the genetic code [8,9]. Cognate amino acid corresponds to the anticodon triplet of the tRNA according to the genetic code. aaRS is the only enzyme capable of implementing the genetic code [10,11]. Accordingly, the aaRS prevents translation errors in the case of incorporating the wrong non-cognate amino acid using hydrolysis through deacetylation activity. As a result, the basal level of translational errors is about 10^−4^ amino acids [12]. 

Among the DNA molecules, the genetic code, mRNAs, tRNAs and aaRSs, only the SSyGC table has physicochemically symmetrically grouped codons for all individual amino acids, including the sextets serine, arginine and leucine. One aaRS is responsible for each amino acid, and in this way, only aaRSs and the genetic code recognise all codons corresponding to a single amino acid. 

Due to the physicochemical similarities of certain amino acids, some aaRSs must rely on additional proofreading mechanisms to prevent tRNA misaminoacylation [13]. According to our research, these additional mechanisms are symmetries of the genetic code (see Section 4). As such, the symmetries are not purely mathematical constructions but also represent physicochemical categories for differentiating codons of the SSyGC table on the principle of purines and pyrimidines of Watson–Crick pairing. Based on the same principle of symmetries, the aaRSs can distinguish codons for its amino acid.

**Figure 2:**
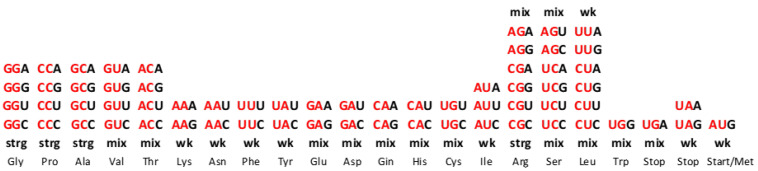
The first two bases (red) determine the position of each codon from split or non-split boxes of amino acids with two or four codons in the genetic code. The characteristics of the first two bases from codons in each box are as follows: (a) strong (strg) CC, GG, CG, GC → non-split boxes because of two strong bases; (b) weak (wk) AA, UU, AU, UA → split boxes because of both two bases; (c) a mix of weak and strong bases AC, UC, GU, CU → non-split boxes because the second base is pyrimidine; (d) a mix of weak and strong bases AG, UG, CA, GA → split boxes because the second base is purine. The sextets serine, arginine and leucine include one non-split box (4/6) and a half of the neighbouring box with two codons (2/4). The purine–pyrimidine characteristics of the first two bases are very important for the differentiation of codons and translation from mRNA to anticodon tRNA. Namely, the first and second bases in mRNA always have canonical pairing in the process of proteinogenesis. Only the third base can have a wobble pairing. One amino acid with two codons from the same box of genetic code has a third base of purines A and G, and the other amino acid pyrimidines U and C. The third base purines A and G have start/stop signals and tryptophan with only one trinucleotide.

**Figure 3:**
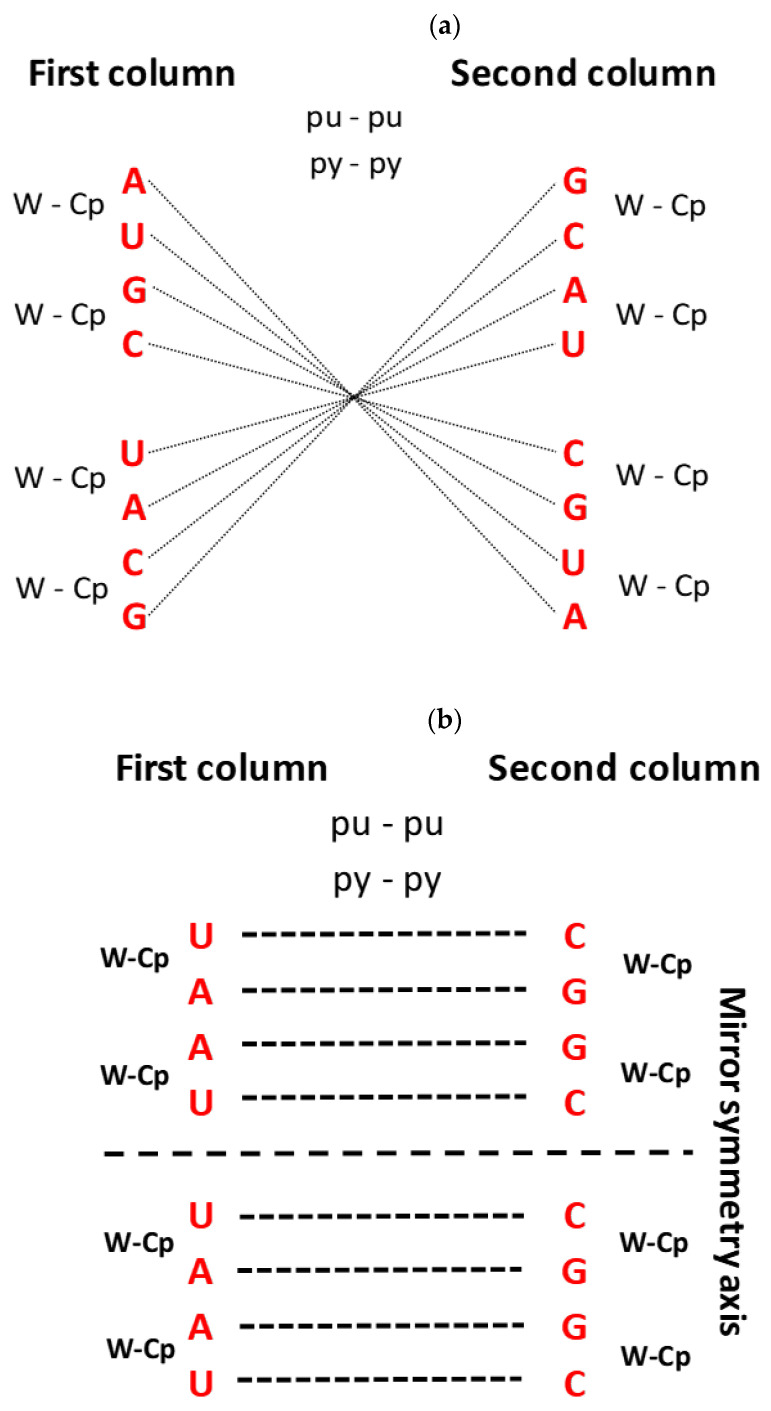

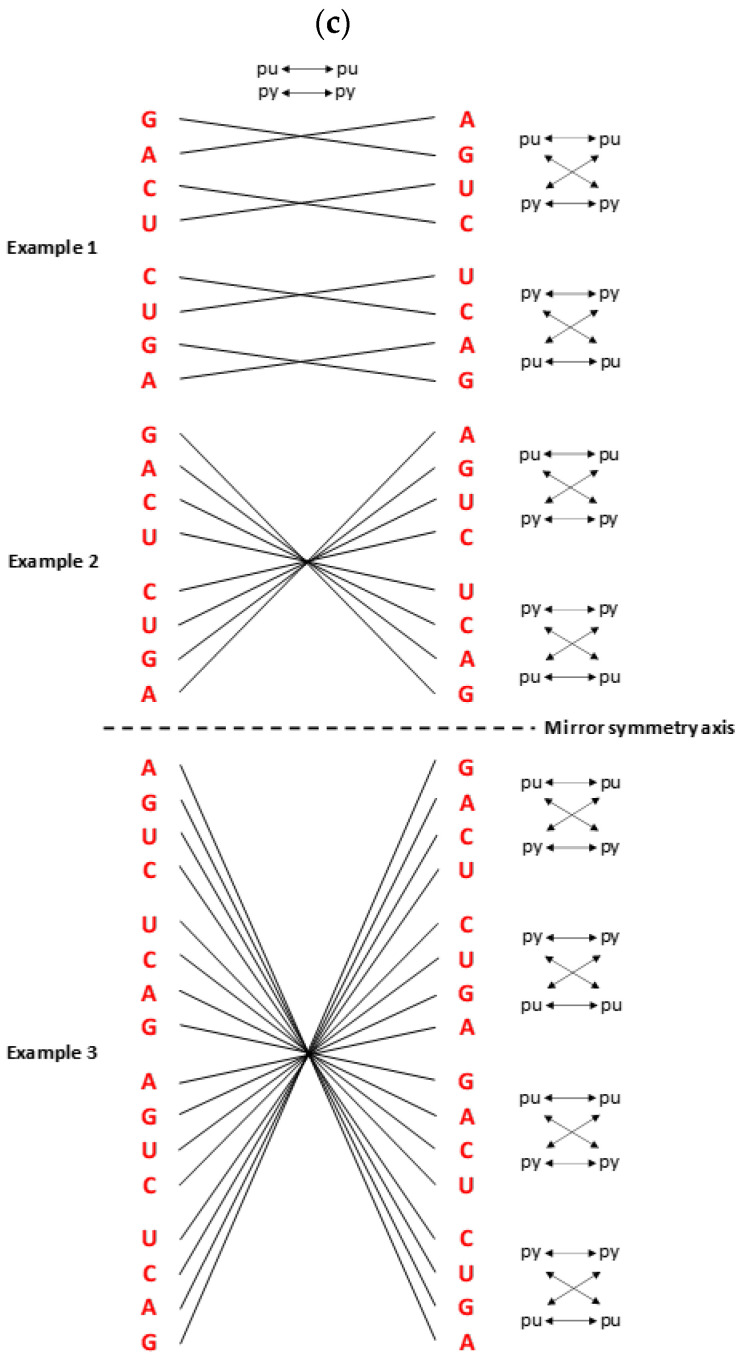
Specific symmetries of each base from codons of the genetic code. (**a**) The centre point of mirror symmetry for the first base of codons in the SSyGC table. Equal first bases in the opposite pair of boxes on each symmetry line passing through the centre point. The first bases point to the composing principle of codon boxes in the whole SSyGC table (in each box, all four codons have identical first and second bases): first bases from a pair of boxes in the vertical direction have Watson–Crick pairing (W-Cp); a pair of boxes in the horizontal direction has purine–purine (pu-pu) and pyrimidine–pyrimidine (py-py) pairing. (**b**) The second base of codons in the SSyGC table. Each pair of bases in the vertical direction is in the form of Watson–Crick base pairing (A↔U, C↔G), and in the horizontal direction, shows purine–purine (G↔A) and pyrimidine–pyrimidine (C-U) pairing. Both halves of the code are identical according to the horizontal symmetry axis. It is very important to observe that the second bases in the whole left column are weak A and U, and in the right column are strong C and G. Due to this separation, the important rule of the codon second base in the differentiation of the amino acids with two or four codons in the flow from mRNA to tRNA is emphasised, which is important for the translation process, especially wobbling pairing in proteinogenesis (see the text). (**c**) The centre points of cascade mirror symmetry for the third base of codons in the SSyGC table. All connecting lines between the same bases in the left and right columns pass through the centre point of the 1st, 2nd and 3rd examples of mirror symmetry. Each cascade mirror symmetry comprises all third bases of the whole genetic code. Additionally, each pair of bases in the vertical and horizontal directions is in the form of purine–purine (G↔A) and pyrimidine–pyrimidine (C↔U) pairing. The third bases of the vertical pairs of boxes have Watson–Crick pairing (G-A-C-U → C-U-G-A and A-G-U-C → U-C-A-G) and the newly discovered purine–pyrimidine symmetries of the SSyGC table. Based on these characteristics, it was possible to mutually distinguish all amino acids and start/stop signals as well as all three sextets (See also Figure 2 and Figure 4).The Standard Genetic Code table ignores the third base of codons because all 16 boxes had an identical U-C-A-G alignment of the third base, and so it cannot show Watson–Crick (codon–anticodon) symmetries between bases and codons (see Section 4).

The most important characteristic of the SSyGC table should be stressed: due to symmetries, each codon has a strictly defined position within the genetic code. The important role have the first and second bases of each codons (Figure 2). It is well known that in each box of twelve bases, the first and second bases are the same for all four codons. However, the second base in the SSyGC table in all boxes from the left column is weak A or U (according to Watson–Crick pairing, in the DNA molecules, A and U are joined with two hydrogen bonds). In the right column, the second base is strong G or C (joined with three hydrogen bonds) (Figure 3b). As such, the first two bases in the codon reveal whether it belongs to a non-split box (all four codons belong to one amino acid) or a split box, which contains two amino acids, each with two codons. Namely, each box is divided on the principle that the third base from both codons of one amino acid contains the purines A and G, and the second amino acid pyrimidines U and C. The AUG start signal and UAG, UAA and UGA stop signals, which are all A+T-rich, arise from the half of the box that contains the purine third base A and G. It is important to observe that none of the signals contain cytosine (Figure 2 and see Section 4).

**Figure 4:**
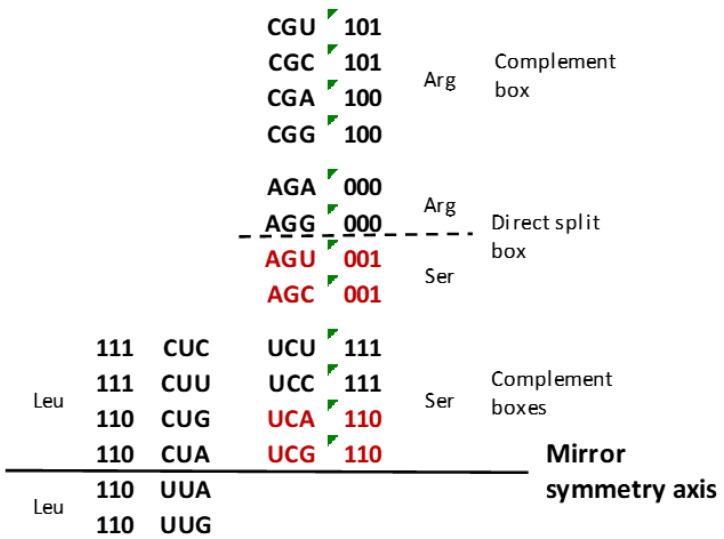
According to symmetries, all three sextets in the SSyGC table have an alignment of codons in continuity: one after another, such as arginine (Arg)–serine (Ser), and one after another, such as serine–leucine (Leu). Only serine has a Watson–Crick relationship of codons between two boxes in the whole genetic code: AGU and AGC are direct—UCA and UCG are their complement (red). Through Watson–Crick pairing, we deciphered the symmetries of the whole genetic code.

According to the characteristics of the first two bases in codons in the SSyGC table, we can also distinguish four groups of codon boxes:

- Non-split boxes with strong bases (GG, CC, GC, CG);
- Split boxes with weak bases (AA, UU, AU, UA);
- Mixed boxes with one strong and one weak base, which are not split because the second base is always pyrimidine (AC, UC, GU, CU);
- Mixed boxes that are split because the second base is always purine (GA, CA, AG, UG) (Figure 2).

Let us consider a few examples. To which amino acid does the codon GUU belong? With respect to the first two bases, the GU codon belongs to the mixed box. Given that the second pyrimidine base is U, this box is not split; therefore, all four codons GUU, GUC, GUA and GUG in this box belong to the same amino acid—valin. 

To which amino acid does the codon UAC belong? The first two bases UA are weak and belong to the split box. The third base is pyrimidine C. Simultaneously, the second codon for this amino acid is UAU, which also has pyrimidine as the third base (UAC, UAU)—they belong to tyrosine.

To which amino acid does the codon UCG belong? The first two bases UC belong to the mixed box, which is not split, because the second base is pyrimidine C. In this example, the amino acid is serine, which belongs to the whole box with UCG, UCA, UCC and UCU codons. Serine is a sextet and in the SSyGC table also has two codons from the neighbouring box. One of these two codons is AGC, where the first two bases are mixed (AG), with purine as a second base, and the box is split. The second codon is AGU, meaning both codons have pyrimidine as the third base (AGC, AGU). Serine shares this box with two other codons, AGA and AGG, with purine as the third base, and they belong to the neighbouring amino acid arginine. 

tRNA is the bridging molecule and the link between mRNA and aaRSs. A cognate amino acid corresponds to the anticodon triplet of the tRNA according to the genetic code. In the cloverleaf structure of tRNA, the basic recognition elements are bases 34, 35 and 36 in the anticodon stem and loop. The canonical translation from an mRNA codon to a tRNA anticodon is bidirectional and under the control of A↔U and C↔G Watson–Crick pairing, i.e., a 3′ codon from mRNA to a 5′ anticodon tRNA relationship. The correct translation ensures that a cognate amino acid is under the control of aaRSs and, as we show, under symmetry control. 

### 2.3. The Relationship between the SSyGC Table and Wobble Hypothesis

The direction of codons in mRNA is 5′3′ due to ester binding between phosphate 5′ and sugar 3′. The third base of the codon has a 3′ end position. The tRNA anticodon has an antiparallel direction with a 5′ end position of the first anticodon base. Namely, it is very important to emphasise that the first and second nucleotides in the codon opposite the second and third nucleotides in the anticodon are always strictly positioned according to canonical Watson–Crick complementary pairing with A↔U and C↔G. Consequently, the second base has the same position in the codon and anticodon. We point out the role of the second base in the codons of the SSyGC table, which reveals which codons belong to amino acids with the whole non-split box of four codons, and which belong to amino acids with two codons each from split boxes. The second base in mRNA codons can have an identical role. This information transfers the tRNA anticodon to aaRSs. When a tRNA reaches an aaRS synthetases bind its amino acid to own acceptor stem t-shaped RNA.

However, the tRNA molecule can recognise a different first base at the 5′ position of the anticodon as wobble pairing, owing to a non-canonical Watson–Crick base pair with the mRNA third base at the 3′ position in the codon–anticodon interaction. In 1966, Crick proposed the wohle hypothesis to explain this partial degeneracy [14]. Namely, there are some differences in the total numbers of codons and tRNAs: for different species, there are on average 40 tRNAs as opposed to the necessary 61 codons and stop signals UAG, UAA and UGA 13]. Wobble pairing partially explains and corrects this disagreement and minimises damage that can be caused by a misreading of the code [13]. 


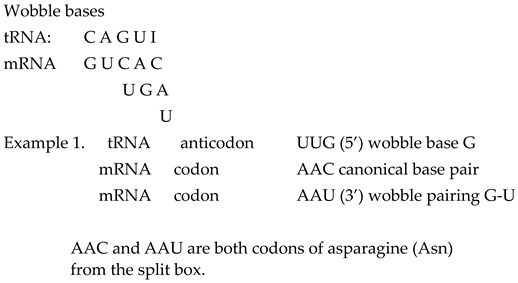


Beside the codon–anticodon canonical Watson–Crick pairing G↔C and U↔A, wobble tolerates pairing between the 5′ G anticodon ↔ 3′ U codon in amino acids with two codons and a third-base pyrimidine C and U from the split box.

From the tRNA anticodon UUG, canonical pairing produces the codon AAC in mRNA, while wobble pairing produces AAU. We show that the two first bases being weak (AA) in the codon reveals the split box, which contains two codons for different amino acids (Figure 2). In this example, the canonical codon is AAC with pyrimidine C as the third base. However, the wobble allows a pairing with AAU, which also has pyrimidine U as the third base, and both codons belong to the same amino acid, asparagine. Due to the symmetries, wobble pairing with the anticodon UUG recognises a second codon, except the regular canonical, from its own amino acid, asparagine. In this way, the message from the RNA is correct, and aaRSs for asparagine can recognise codons for its amino acid and link tRNA-appropriate amino acids into a growing polypeptide chain. The combination tRNA G → mRNA U, through wobble pairing with only one anticodon, solves the problem regarding an amino acid with two codons, both of which have pyrimidine (U, C) as the third base.


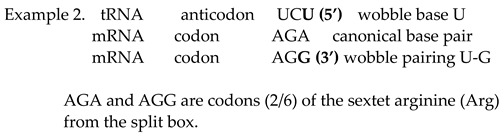


The tRNA anticodon UCU has a canonical pairing with codon AGA on mRNA with a mixed first two bases (AG), but the second base is purine, and, therefore, the box is split (Figure 2). This amino acid is arginine with two codons (2/6) from the split box. In other words, the canonical codon is AGA with purine (A) as the third base. The wobble allows a pairing with AGG, whose codon also has purine (G) as the third base. AGG and AGA are both codons (2/6 from the split box) for the amino acid arginine, and the message will be correctly recognised by aaRSs. In this example, the combination anticodon tRNA U → codon mRNA G through wobble pairing solves the problem regarding amino acids with two codons from the split box that have purine (A, G) as the third base. The message from tRNA is correct, and aaRSs for the two codons from the split box of arginine can recognise the codon for its amino acid and link the tRNA-appropriate amino acid into a growing polypeptide chain. The remaining four codons (4/6) from the non-split box have wobble pairing with inosine (see example 3).


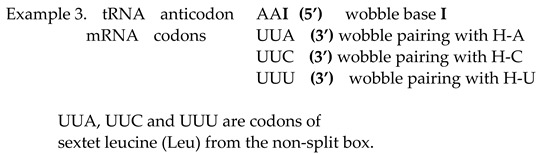


Only tRNA can have inosine as the first base (5′) on the anticodon. The nucleobase of inosine is hypoxanthine. There are three types: hypoxanthine adenine (H-A), hypoxanthine cytosine (H-C) and hypoxanthine uracil (H-U). Inosine in the position of the first base in the anticodon can be matched with three bases (A, C and U) as the third base (3′) in the codons of mRNA. The wobble with inosine is suitable for amino acids that contain four codons of the non-split box, and the wobble pairing is possible with each of the bases A, C and U. In non-split boxes, the third base G of the codon from mRNA always has canonical pairing with the anticodon base C from tRNA. 

In our example, the tRNA anticodon AAI has wobble pairing with codons that have the first two weak bases UU, but the second base is pyrimidine U; therefore, this amino acid is from the non-split box and has four codons (Figure 2). A wobble with inosine enables pairing with UUA, UUC and UUU codons in mRNA. The message for all three codons will be appropriate because it belongs to the same amino acid, which will be recognised by aaRSs for leucine. The fourth codon UUG has a canonical Watson–Crick pairing with the AAC anticodon on tRNA. In the genetic code, there are eight amino acids that possess the whole box with four codons, including the sextets serine, arginine and leucine.

These three examples of wobble pairing cover all natural amino acids from the genetic code. It is important to observe that the symmetry modification of the anticodon stem and loop domain on tRNA with wobble pairing includes the positions 34, 35 and 36, with the first base of the anticodon in position 34. Although there are fewer tRNA than mRNA, wobble pairing ensures that not one amino acid remains left out of the translation process.

A question arises: why does only inosine satisfy wobble pairing with an amino acid from the non-split box? See example 1 for the two codons AAC and AAU from the amino acid asparagine from the split box. The wobble pairing with the anticodon inosine UUI in this example could also recognise the AAA codon from the second amino acid, lysine, from each box of mRNA as a misreading error.

Example 2 for the two codons AGA and AGG from the split box of amino acid arginine shows that wobble pairing is possible with the inosine UCI anticodon for the AGA codon. In this example, inosine could also recognise AGC and AGU codons from the split box of the second amino acid serine as a misreading error. We show that wobble pairing with inosine is correct and without misreading, with only eight amino acids, which have four codons from the non-split box.

**Figure 5:**
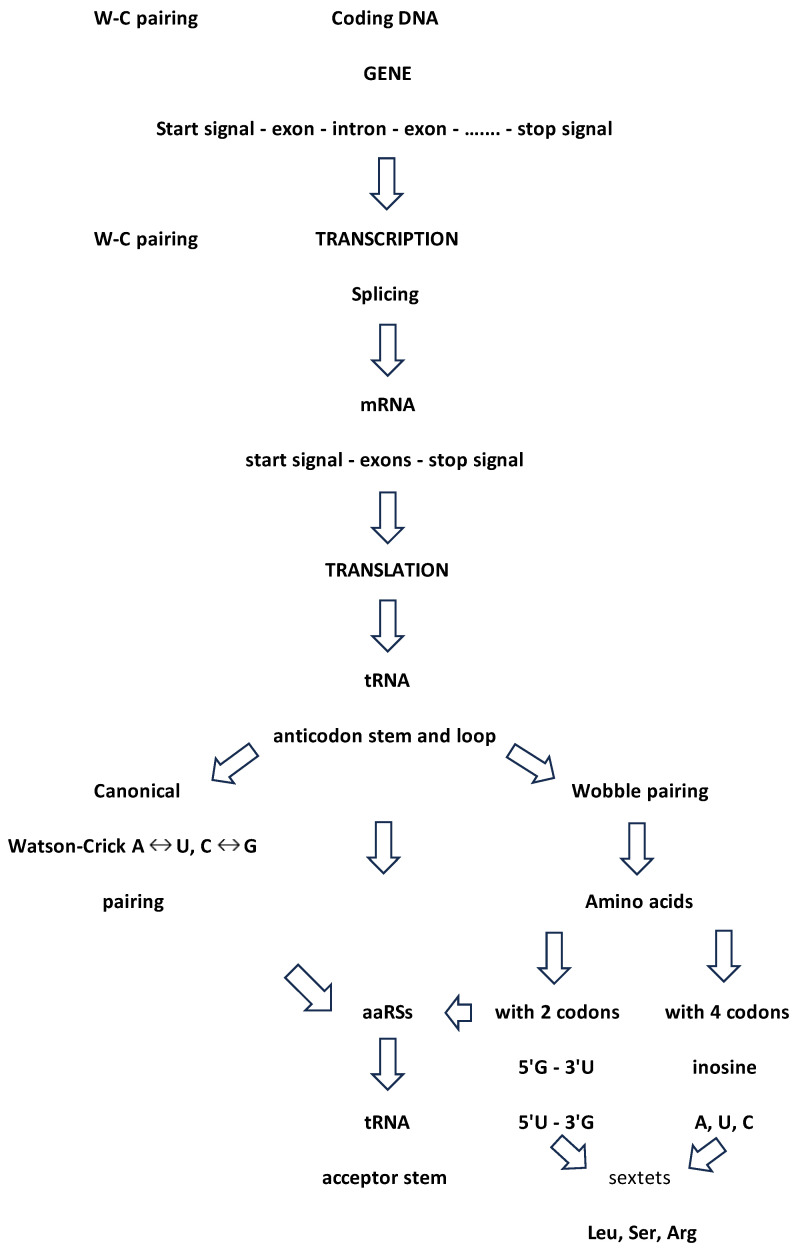
Algorithm of proteinogenesis. All stages from coding DNA over mRNA to tRNA are based on Watson–Crick pairing. The critical point is the process of translation because the number of mRNA codons is larger than the number of tRNA anticodons. As a result, canonical base pairing must be supplemented with wobble non-canonical hydrogen bonding 5′G→3′U or 5′U→3′G for an amino acid with two codons, and inosine A, inosine U or inosine C for amino acids with four codons. In this case, the SSyGC table plays an important role as it reveals the core purine–pyrimidine symmetry net in which each codon has a strictly defined position with respect to other codons. At the same time, each codon with its purine–pyrimidine structure of first and second bases reveals whether it is from an amino acid with two or four codons. The sextets leucine (Leu), serine (Ser) and arginine (Arg) have a combination of both types of wobble pairing.

Errors such as these are prevented due to symmetries, which enable the recognition of each codon, whether it belongs to an amino acid coded by two codons from the split box or by all four codons from the non-split box. We discovered this regularity due to the SSyGC table (Figure 5). 

The discovery of new modifications within the anticodon stem and loop of tRNA, for example, the C32 position and the A37 position, led to the restriction or expansion of the abilities of wobble pairing and enabled the specific recognition of canonical codons [13].

### 2.4. Wobble Pairing in Alternative Genetic Codes

Translation is not limited to twenty amino acids. The additional selenocysteine, as the 21st amino acid, takes the UGA codon and the position of the UGA stop signal, and pyrolysine, as the 22nd amino acid, takes the UAG codon and the position of the UAG stop signal [7]. However, in our SSyGC table, the position of the codons and the basic purine–pyrimidine symmetry net remain unchanged. 

**Figure 6:**
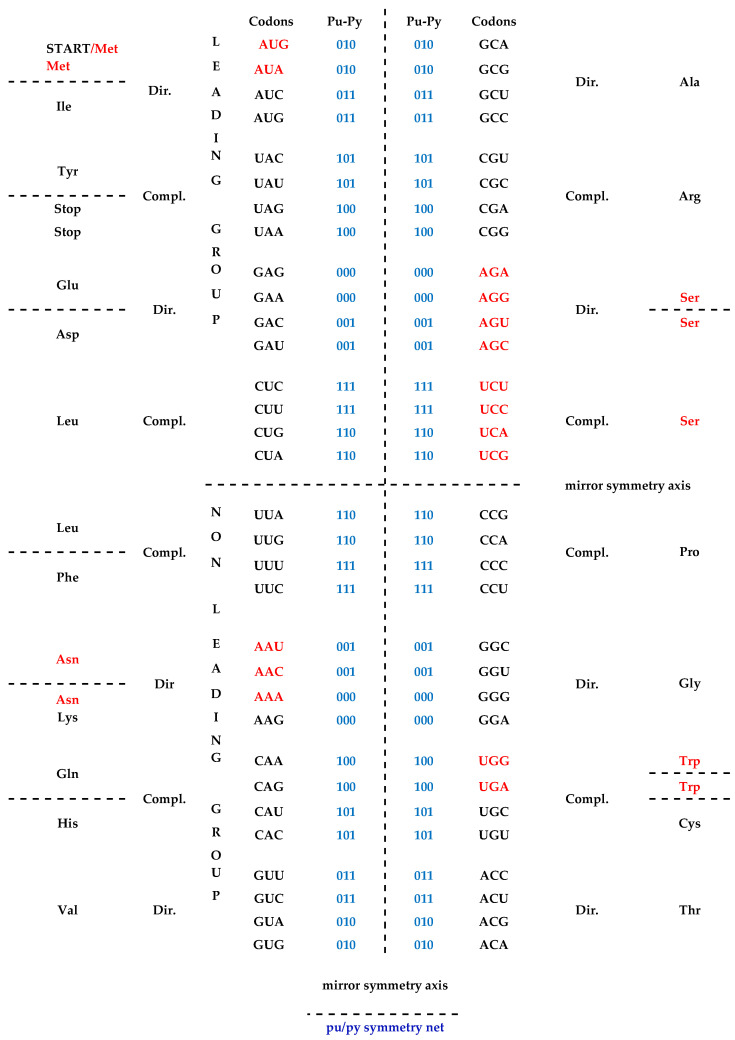
The mitochondrial trematode (liver-fluke) code incorporated in the Supersymmetry Genetic Code table. Methionine (Met) expands to the neighbouring isoleucine (Ile) codon AUA; tryptophan (Trp) expands to the neighbouring stop UGA codon; arginine (Arg) neighbouring AGA and AGG codons become the 7th and 8th codons for serine (Ser); asparagine (Asn) expands to the neighbouring AAA codon from lysine (Lys). In alternative genetic codes, individual amino acids usually capture a codon from a neighbouring amino acid in the SSyGC table. But the purine–pyrimidine symmetry net always remains unchangeable. All (more than thirty) nuclear or mitochondrial genetic codes different from the Standard Genetic Code have also been incorporated into the SSyGC table without interrupting the symmetries. Blue: purine–pyrimidine (pu-py) symmetry net. The leading group, due to the position of serine, led to the discovery of symmetries in the whole SSyGC table (Figure 4).

We show that the SSyGC table, with its physicochemical symmetries including the purine–pyrimidine symmetry net, is common for more than thirty alternative genetic codes. Namely, a variant number of codons for individual amino acids inserted in the SSyGC table arise more often by capture from the neighbouring codons from the split box, while the position of codons in the genetic code remains unchanged. Some mitochondrial codes, such as the trematode code (Figure 6), invertebrate code, echinoderm code, flatworm code and alternative flatworm code with eight codons for serine, which build amino acids in most proteins, participate in the regulation of energy metabolism and fuel storage [7].

The wobble pairing of codons in the translation process of amino acids from the alternative genetic codes is identical to the examples in Section 2.3. Let us see the mitochondrial trematode (liver-fluke) code incorporated into the Supersymmetry Genetic Code table. Methionine usurps the neighbouring isoleucine codon AUA: 

         
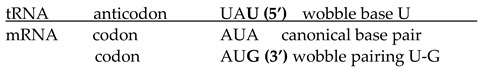


AUA and AUG are both codons of methionine in the split box (AU are weak bases). Anticodon UAU with a wobble base U can recognise its canonical codon and wobble pair AUG. In the nuclear genetic code, AUG is a single codon for the start signal and methionine. If AUG is in front of the gene, it is a start signal, and if it is inside the gene, it is a codon for methionine. It only has a canonical translation between the mRNA codon AUG (3′) and the tRNA anticodon UAC (5′).

Asparagine usurps the AAA codon from lysine, and now it has three codons from the split box (AA are weak bases): AAU, AAC and AAA. The wobble pairing from AAU and AAC codons is example 1, and the AAA codon has canonical pairing with the anticodon UUU. The wobble pairing in this case, with inosine as the first base (5′) in the anticodon, cannot be matched with all three bases A, C and U as the third base (3′) in the AAU, AAC and AAA codons of mRNA because the second base is purine A in the split box. Inosine can recognise codons from the non-split box with a pyrimidine second base. 

Serine usurps both codons (AGU, AGC) from arginine from the split box. The wobble pairing is example 2. aaRS is the only enzyme capable of implementing the genetic code because it distinguishes all its own codons, in this example, all eight codons for serine. 

Tryptophan with only one C+G-rich codon UGG usurps a neighbouring UGA stop signal. The wobble pairing is example 1, but UGA alone as a stop signal terminates translation by binding to a release factor (see next subsection).

### 2.5. The Wobble Pairing of Sextets Serine, Leucine and Arginine

**Figure 7:**
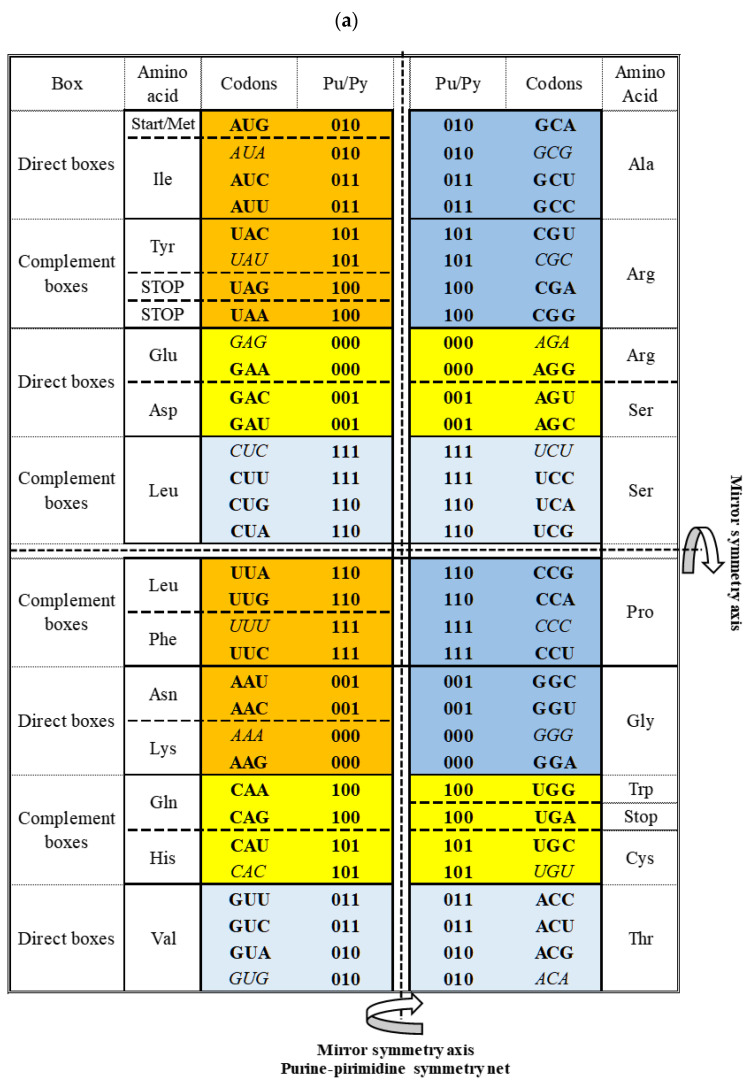

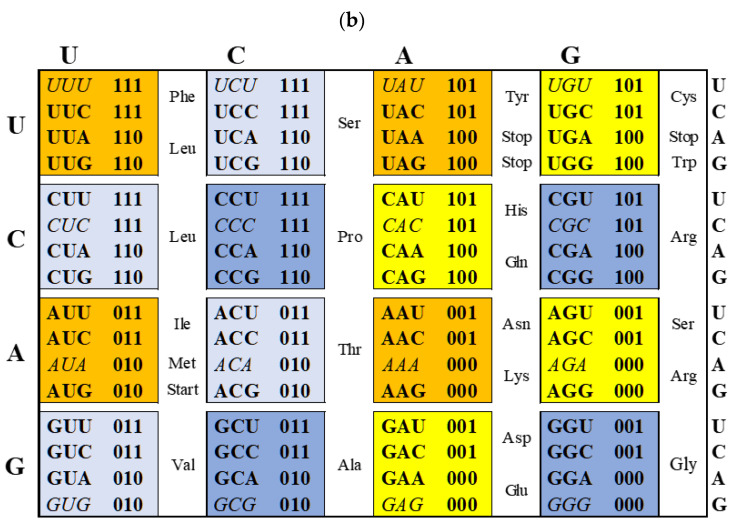
The difference between the symmetry structures of the Supersymmetry Genetic Code (SSyGC) table and the Standard Genetic Code table. (**a**) The SSyGC table incorporates 2 × 8 boxes with four codons in each box and starts with an AUG initiation start signal. Only the SSyGC table has in continuity the codons of three amino acids with six codons each: serine, arginine and leucine. The SSyGC table has the same distribution of the purine/pyrimidine profile in both columns, and simultaneously the same profile distribution pairs of codon rows within each box. With horizontal and vertical central mirror symmetry axes, it creates the purine–pyrimidine symmetry net as “the golden rule” for all RNA and DNA living species. Between both columns in the same row, there are alternate transformations of A+U-rich and C+G-rich codons. There is a symmetrical position between the split and non-split codon boxes. The symmetries of the genetic code mean that each codon has a powerful, specific symmetrical position in the SSyGC table. This can be observed, for example, in the symmetrical position of the symmetrical codons (in italics). 0 pu, purine; 1 py, pyrimidine; dark yellow, two pairs of split boxes with direct–complement symmetry between codons; dark blue, two pairs of non-split boxes with direct–complement symmetry between codons; light yellow, two pairs of split boxes with purine ↔ purine, pyrimidine ↔ pyrimidine transformation between codons; light blue, two pairs of non-split boxes with purine ↔ purine, pyrimidine ↔ pyrimidine transformation between codons. (**b**) The Standard Genetic Code incorporates 4x4 boxes, also with four codons in each box. Codons are positioned alphabetically in a horizontal and vertical array of U-C-A-G bases. The role of the third base of codons was ignored in the search for the Standard Genetic Code symmetries because the U-C-A-G arrays were the same in all boxes. The second base of codons divides the standard code into two halves, with pyrimidine (U, C) symmetries and purine (G, A) symmetries. However, these base halves do not communicate with each other on the principle of physicochemical symmetries as in the SSyGC table. The colour is identical in boxes with the same codons as in the SSyGC table in (**a**), but neither boxes nor codons have an identical position to in the SSyGC table.

The SSyGC table follows in continuity all three sextets, one under another (arginine–serine) or one to another (serine–leucine), and each includes a whole box with four codons and half of the neighbouring box with two codons (Figure 4 and Figure 7a). Based on this principle, serine has a unique Watson–Crick pairing in the context of the whole SSyGC table between its AGU, AGC↔UCA and UCG codons from the split box and four codons from the non-split neighbouring box. This codon–anticodon (direct–complement) construction helped us discover and recognise the positions of and symmetries between all codons from the SSyGC table.

In the horizontal direction, leucine arises from purine–purine and pyrimidine–pyrimidine transformation from serine. Its two codons from the neighbouring non-split and split boxes (C**UG**, C**UA**↔U**UA**, U**UG**) have identical second and third bases.

In the vertical direction, arginine arises from serine, and its two codons from non-split and split boxes (C**GA**, C**GG**↔A**GA**, A**GG**) also have identical second and third bases. 

From serine, tRNA can recognise all four codons from the whole box (UCU, UCC, UCA, UCG) due to the UC mix of first and second bases, but with pyrimidine as a second base—this box is non-split (Figure 7a). At the same time, tRNA can also recognise the two codons (AGC and AGU) of serine from the split box, namely, due to the AG mix of first and second bases, but with purine as the second base—this box is split.

From leucine, tRNA recognises that all four codons (CUC, CUU, CUG, CUA) have two weak UU first and second bases and they are from the split box (Figure 7a). 

tRNA can recognise all four codons (CGU, CGC, CGA, CGG) of arginine. They are from the non-split box due to the strong CG of the first and second bases. The remaining two codons (AGA and AGG) divide with two codons of serine from the split box because of the mix of AG first and second bases but with purine as the second base (Figure 7a).

In regard to the symmetrical organisation of the SSyGC table, each aaRSs can recognise all codons of its amino acid.

We show that all amino acids with four codons from the non-split box can realise wobble pairing with inosine without a misreading error. This is also valid for four codons of non-split boxes from serine, leucine and arginine (see example 3, Section 2.3). Sextets can also carry out wobble pairing for amino acids with two codons according to the third base on mRNA if it is pyrimidine on the principle of anticodon G 5′ → codon U 3′ (see example 1), as for two codons for serine, and if it is purine on the principle of anticodon U 5′ → codon G 3′ (see example 2), as for two codons for leucine and arginine.

### 2.6. The Differentiation between All Start/Stop Signals 

One characteristic that the start AUG and three stop UAG, UAA and UAG signals have in common is that they contain A+U-rich trinucleotides. However, none of the signals have cytosine, and the third base is purine G and A. The stop signals terminate translation by binding to release factors rather than tRNA molecules [13].

The purine–pyrimidine structure of all three stop signals is 1-0-0 (1 pyrimidine, 0 purine), and they are A+U-rich. Stop signals UAG and UAA have the first two weak bases U and A, and stop signal UGA has mixed bases, with the second base being purine (G) (Figure 2). Accordingly, tRNA can recognise that they are from split boxes. In the SSyGC table, there are eight codons of the same 1-0-0 purine–pyrimidine structure: UAG, UAA and UGA are stop signals, and the others (CGA, CGG, CAA, CAG) are codons that contain cytosine (Figure 7a). The partner of the stop signal UGA in the same half of the box is AGG (codon for tryptophan), which is C+G-rich. Therefore, at any level of the genetic code, all three stop signals are differentiated on the grounds of the purine–pyrimidine symmetrical structure.

The start signal AUG, as the initiation codon for genes, is also at the beginning of our SSyGC table. It is an A+U-rich signal with the purine–pyrimidine structure 1-0-1 from split boxes. Its neighbour is also an A+U-rich weak symmetric codon, AUA, with the third base purine as well as the AUG start signal (or codon for methionine) from the same split box. Therefore, it is possible that, in rare cases, AUA transforms in the second codon for methionine. The remaining trinucleotides of the same purine–pyrimidine 0-1-0 structure are GCA, GCG, GUA, GUG, ACG and ACA, which belong to non-split boxes because they have the second base pyrimidine. Accordingly, the top position of the start signal is unique in the SSyGC table and recognisable due to purine–pyrimidine symmetries. 

### 2.7. The Universality of Genetic Code

It is necessary to explain a very important aspect of the SSyGC table—the universality. This means that each codon has a strictly determined position in the genetic code, which cannot be exchanged with any other codon. In 2019, J. Fredens and coworkers performed an important experiment [15]. They created *Escherichia coli* as a laboratory “synthetic” species with the entire synthetic DNA genome that utilised 59 codons and two stop signals to encode 20 amino acids for protein synthesis, compared to 61 codons and three stop signals in natural living organisms. *E. coli* had an evolutionary path whereby it started by itself in the early beginning of the origins of life of living species on Earth. The number of bases in the genome of *E. coli* is about 5 million, for example, only in a centromere of a human chromosome, and it also has all 20 natural amino acids. By performing “synonymous codon compression”, the authors recompiled the *E. coli* genome but omitted two (UCG, UCA) out of six codons encoding serine and a UAG stop signal. They concluded that *E. coli* with “synthetic DNA” displayed only minor changes, with a slower growth rate and slightly elongated cells, which enabled the deletion of previously essential tRNA. However, the attempts at changing other amino acids were not successful; i.e., *E. coli* did not survive. 

Here, we highlight the important role of serine. Note that the experiment succeeded—*E. coli* survived—if the two codons UCG and UCA were removed just from serine (leaving their complements AGC and AGU in the remaining four codons). The replication of a DNA molecule means that each strand can be reconstructed based on the principle of Watson–Crick pairing (A↔U, C↔G) from the opposite non-mutilated strand. It means that *E. coli* could be reconstructed successfully with AGC and AGU complements for serine, returning its complete genome. This experiment showed that of crucial importance is a balanced need for all six codons of serine, which is vitally responsible for energy demand. 

The removed codons and stop signal from *E. coli* could not be replaced in the purine–pyrimidine symmetry net by other codons, and that partially reconstructed genome resulted in a mutant with a changed phenotype and a slower growth rate. In this way, the experiment with *E. coli* did not prove that a new synthetic species with an unnatural genetic code was created. On the contrary, it proved an important fact that, for the normal development of species, a complete genetic code with 20 natural amino acids is needed, structured on the basis of purine–pyrimidine physicochemical symmetries. Violation of symmetries endangers species and creates mutants, while the omission of codons for an individual amino acid endangers the life of species.

According to the experiment with *E. coli*, it can be concluded that the different number of codons for each amino acid depends on the metabolic necessity of the individual species. At the same time, the codons for the individual amino acids in mRNA are mutually equivalent. However, during translation, there is a differentiation between codons with canonical tRNA anticodon pairing and wobble pairing; namely, because of the unequal number of codons and tRNA anticodons, wobble pairing is activated. We show that this process of mRNA–tRNA translation helps the purine–pyrimidine symmetries of the genetic code to recognise codon–anticodon pairing as well as which codon belongs to an amino acid with two codons and which belongs to an amino acid with four codons to realise optimal wobble pairing (Figure 4). Accordingly, misreading during the process of translation in proteinogenesis is exceptionally rare and is reduced to only 10^−4^. 

## 3. Discussion

The genetic code regulates how the translation system decodes the 61 genetic codons into 20 natural amino acids along with three termination signals in the process of proteinogenesis. The genetic code is degenerate because more than one type of codon encodes a single amino acid. In general, degeneracy is associated with the mathematical group theoretical structure [16,17,18,19,20,21]. An algebraic approach to the SGC was proposed with the aim of explaining the degeneracies encountered with a sequence of symmetry breaking. However, our understanding of the evolution of the genetic code through progressive symmetry breaking, when using the group theoretical structure, holds that, in the beginning, it was not possible to distinguish the functions of codons as they all encoded the same information. With the consecutive creation of amino acids during such a proposed evolution, the number of codons within the degeneracy groups gradually decreased into two singlets (methionine/start signal and tryptophane), nine doubles, two triplets (isoleucine and three stop signals), five quadruplets and three sextets. 

The degeneracy distribution considers only the number of codons for each amino acid according to Nirenberg’s empirical results, without including any physicochemical affinity between bases and codons [7]. However, the degeneracy of amino acid coding takes place for the most crucial and enigmatic aspects to this day. For example, some authors have a new theory based on symmetry principles of code degeneracy, hypothesising that the primitive pre-early code had codons of four bases. Only 32 codons of 256 possible combinations (4^4^) had some symmetries with amino acid degeneracy 2, and the rest were asymmetric codons with amino acid degeneracy 4 [22]. This mathematical model is also based on number theory, starting from extant variants of the last universal common ancestor (LUCA), which was the first to have the universal genetic code with three-nucleotide codons [22,23,24,25] like the present standard genetic code.

Despite genetic code degeneracy, all symmetries, including double mirror symmetry and the purine–pyrimidine symmetry net of the SSyGC table, remain unchanged for all RNA and DNA living species (Section 2.7, Figure 3 and Figure 7b). 

Negadi, 2023 [26], presented a novel approach to studying the genetic code’s mathematical and chemical structure, revealing the genetic code symmetries through computations involving Fibonacci-like sequences. They found a full mathematical and chemical connection with the “ideal sextet’s classification scheme” of our Ideal Genetic Code table [27]. Moreover, they examined the total number of hydrogen chains in all amino acids’ 61 sense codons from the Ideal Genetic Code table and proposed “the hydrogen and atoms numbers as “seeds” of three sextets (serine, arginine and leucine), which will create the entire hydrogen atom and even nucleon content of the whole set of amino acids and will also play a prominent role mathematical and (chemically inspired) “seeds” in computing the chemical content of the twenty amino acids, including degeneracy”. The same author, in 2024 [28], used Fibonacci-like sequences to examine the symmetries of our Supersymmetry Genetic Code table, derived from the Ideal Genetic Code table [6], in a charged physiological state of amino acids in a pH environment around 7.4, testing the efficiency of his method of using the atom content in unravelling relationships with the genetic code, with vertical and horizontal mirror symmetry axes between all purines and pyrimidines of the whole code. 

Breslauer et al., 1986 [29], and Klump et al., 2020 [30], measured the free energy of codons using spectroscopic and calorimetric techniques and concluded that the free energy of each codon is equal to the free energy of its reverse complement. When they inserted these values in the mitochondrial human genetic code table, they were randomly scattered. The authors analysed the free energy of codons in the SGC table. We show that the mirror symmetry of the SSyGC table, DNA quadruplets and our classification of codons and trinucleotides is perfectly imbedded in the mirror symmetry energy code, as well as having identical mirror symmetry to the free energy values for all four members of the DNA quadruplets and codon/trinucleotide quadruplets of their classification [31]. 

Lei Lei and Burton, 2023 [32], observed that features of the genetic code model include explanations for the coevolution of amino acid metabolism, amino acid chemistry, homologous aaRS enzymes, tRNAomes and stop codons. The author stated, “wobbling at tRNA-34 evolved as the ribosome “learned” to read the anticodon”. The authors analysed the SGC without mentioning symmetries, but we have shown that symmetries have an important role in wobbling translation.

The main problem as to why Crick’s Standard Genetic Code table does not reveal physicochemical symmetries is its organisation with vertical and horizontal U-C-A-G alignments of codons. The second base of codons divides the standard code into two halves: one with pyrimidine (U, C) symmetries and the other with purine (G, A) symmetries (Figure 7b). However, these halves do not communicate with each other on the principle of physicochemical symmetries as in Watson–Crick pairing, codon–anticodon or purine–purine and pyrimidine–pyrimidine pairing.

Contrary to this, our SSyGC table is completely organised on the principle of physicochemical purine–pyrimidine symmetries of bases and codons, as well as of start/stop signals, meaning it is not only a numerical observation that features the genetic code. 

Given the double mirror symmetry of the purine–pyrimidine symmetry net, it is not a coincidence that the SSyGC table starts with an AUG start signal. It is also not a coincidence that three sextets are aligned in continuity in the SSyGC table: serine, arginine and leucine. On the same principle, all codons for amino acids are strictly symmetrically distributed within the SSyGC table, and each amino acid has its unique and recognisable “territory” inside the code. This is seen, for example, in the positions of the three stop signals. With their 1-0-0 organisation (purine 1, pyrimidine 0), all start/stop signals have A+U-rich bases and do not contain cytosine. Therefore, they are symmetrically arranged within the SSyGC table (Figure 7a).

According to our classification of codons/trinucleotides [7], we also show the purine–pyrimidine symmetry between A+U/T-rich and C+G-rich codons/trinucleotides.

Our “symmetry-based theory of the genetic code” with the SSyGC table also supports the role of symmetries in the processes of transcription and spatial translation. We show that the same symmetries contained in the genetic code play a fundamental role in the recognition and differentiation of codons from DNA, mRNA, tRNA and aaRSs in transcription and translation during proteinogenesis. The genetic code and aaRSs play a key role in these processes, because they have information about the type and number of codons for each amino acid. Symmetries enable tRNA transfer and correct information about the individual codon from mRNA in the creation of the amino acid chain. 

According to the symmetries of the genetic code based on the purine–pyrimidine structure, it is possible to determine the following: (1) which codon belongs to a particular amino acid, (2) whether there is an amino acid that contains the whole box with four codons or an amino acid with only two codons, (3) if there are weak or strong codons and (4) whether there are A+T-rich or C+G-rich codons. One can find all the biological physicochemical symmetries in addition to mathematical quantities [26,28]. We show that, due to the physicochemical purine–pyrimidine symmetries, all these characteristics are translated to aaRSs, which can recognise all codons from their own amino acid. 

We also show that, due to symmetries between codons in the genetic code, wobble pairing during the translation of codons from mRNA to the anticodon tRNA does not violate the structure and organisation of codons for an individual amino acid, despite not satisfying Watson–Crick U↔A, C↔G base pairing. Therefore, it does not lead to misreading, and the errors during translation are reduced to only 10^−4^.

In many previous publications, we have not found it mentioned that symmetries might play a role in transcription and translation. This is probably due to the late discovery of genetic code symmetries in 2022 [6]. It is interesting that the same symmetries also support the wobble hypothesis. We show that the purposeful use of inosine is for amino acids from non-split boxes with four codons. Namely, inosine is not the natural base of mRNA codons but only in the anticodon tRNA. On the contrary, for the two amino acids from the split box with only two codons each, wobble pairing takes into consideration purines or pyrimidines as third bases of the codon with canonical and wobble pairing from the same amino acid.

In conclusion, we can say that the hypothesis regarding the development of amino acids that evolved through wobble and super wobble in the Standard Genetic Code [22] or through the primitive genetic code and the early genetic code, only from LUCA evolution, is at the present time based on a complete genetic code for all twenty natural amino acids with trinucleotide codons [23,24,25]. We have shown that in the context of this long evolutionary period, the SSyGC table is a realisation of purine–pyrimidine symmetries based on the principle of physicochemical laws, common and unchangeable for all RNA and DNA living species [6,7,33]. The symmetries of the genetic code applied to the transcription and translation of proteinogenesis highlight the role of physicochemical purine–pyrimidine symmetries in decreasing disorder with error minimisation and in preserving the integrity of the biological system. Only purine–pyrimidine symmetries with a central role of the genetic code apply to the whole system and genetic information flow from DNA to proteins. Recalling again the beginning with Einstein’s principle of symmetry in physics as the primary feature of nature, we have extended this principle to fundamental biological processes such as DNA and the genetic code and to the origin of life. In any further research, symmetries of the genetic code cannot be ignored.

## 4. Materials and Methods

### The Difference between the Standard and Supersymmetry Genetic Code Tables 

The SGC table incorporates 4x4 boxes with four codons in each box. The codons are positioned alphabetically in a horizontal and vertical array of U-C-A-G bases (Figure 7b). The role of the third base of codons was ignored in the search for genetic code symmetries because the U-C-A-G array was the same in all 16 boxes. Accordingly, the codons in each box were differentiated only according to the first two bases.

Regardless of the non-random structure of the SGC table, much effort was directed towards finding symmetries in the genetic code. For a long time, complete symmetry of the genetic code had not been found, leaving doubt as to whether a symmetrical nature as the protector of order even existed. Crick proposed a solution to the problem of genetic code evolution with the random “frozen accident hypothesis”, concerning the code’s inability in one stage to accept new variations [5]. Almost 50 years after the discovery of the genetic code, Eugen V. Koonin in 2009 postulated the stereochemical theory according to which “codon assignments are dictated by a physicochemical affinity between amino acids and the cognate codons/anticodons. The specific structure of the code is not at all accidental but, rather necessary and, possibly, unique”. Koonin was sceptical about the potential for a quick discovery and understanding of the origin and evolution of the genetic code [19].

Our genetic code is structured from purine–pyrimidine codon symmetries for 20 natural amino acids. Therefore, symmetries of the genetic code were sought predominantly between purines (A, G) and pyrimidines (U, C) of codons and amino acids follow this direction. 

Based on the fundamental symmetries of our SSyGC table, we postulate “the symmetry theory of the genetic code”. Due to the complete physicochemical symmetries of the SSyGC table with the unique purine–pyrimidine symmetry net, it is not necessary to involve “the frozen accident” hypothesis.

We have shown the multifaceted symmetries of the SSyGC table (Figure 7a) as follows:

- Only the SSyGC table with 2 × 8 boxes accounts for the whole richness of the symmetries.
- The SSGC table starts with the AUG initiation codon, also known as the start signal.
- Only the SSyGC table has all codons of three amino acids with six codons each (serine, arginine and leucine) ordered in continuity (Figure 4).
- Across the whole genetic code, in both columns the same purine (0)–pyrimidine (1) relationship of codons appears.
- In each horizontal pair of boxes, there are pairs of rows with the same purine–pyrimidine relationship.
- There are direct–complement/codon–anticodon (5′3′–3′5′)/Watson–Crick pairing relationships between codons in each vertical pair of boxes.
- The SSyGC table with its Watson–Crick pairing between bases and codons can be directly transformed into a DNA molecule with the direct codons for one DNA strand and the complement codons for the opposite strand (Figure 1).
- In the middle of the code, the unique purine–pyrimidine symmetry net with the double mirror symmetry is positioned according to the vertical and horizontal mirror symmetry axes (Figure 6 and Figure 7a).
- It is very important to point out that the symmetry net as “the golden rule” is unique for all RNA and DNA species and unchangeable during evolution.
- In each row of the code, we emphasise purine–purine and pyrimidine–pyrimidine transformation A+U-rich (week) ↔ C+G-rich (strong) codons. Namely, weak A and U bases have two H-bonds between the DNA strands, and strong C and G bases have three H-bonds.
- In the left column of the SSyGC table, A+U-rich codons (24/8) are dominant, while in the right column, C+G-rich codons (24/8) are dominant.
- In the whole genetic code table, A+U-rich and C+G-rich codons are regularly mutually arranged, although not with mirror symmetry.
- In the SSyGC table, there is a regularity between the number and position of the split and non-split boxes.
- We can clearly see a symmetrical distribution of the individual bases of each codon across the whole SSyGC table (Figure 3).
- The third codon bases of the whole genetic code each comprise cascade mirror symmetry (Figure 3c).
- The split or non-split boxes have a third base of codons separate on two purines (A, G) and two pyrimidines (U, C).
- All boxes in the left column of the SSyGC table have a codon with a U or A weak second base, and all boxes in the right column have a codon with a C or G strong second base, which is very important for translation (Figure 3b).

In conclusion, we can say that the structure of the SSyGC table is based on physicochemical purine–pyrimidine symmetries between bases and codons. The position of each codon in the genetic code is strongly defined and unchangeable with another codon. Violation of symmetries endangers species and creates mutants, while the omission of codons for an individual amino acid endangers the life of the species [14,20].

## Data Availability

We processed the relevant and original data of purine–pyrimidine as well as the codon symmetries from our Supersymmetry Genetic Code table in comparison with the alphabetical purine–pyrimidine symmetry of the Standard Genetic Code table.
